# Restricted Mean Survival Time Analysis to Estimate SGLT2i–Associated Heterogeneous Treatment Effects on Primary and Secondary Prevention of Cardiorenal Outcomes in Patients With Type 2 Diabetes in Taiwan

**DOI:** 10.1001/jamanetworkopen.2022.46928

**Published:** 2022-12-15

**Authors:** Zi-Yang Peng, Chun-Ting Yang, Shihchen Kuo, Chih-Hsing Wu, Wei-Hung Lin, Huang-Tz Ou

**Affiliations:** 1 Institute of Clinical Pharmacy and Pharmaceutical Sciences, College of Medicine, National Cheng Kung University, Tainan, Taiwan; 2 Division of Metabolism, Endocrinology & Diabetes, Department of Internal Medicine, University of Michigan Medical School, Ann Arbor; 3 Department of Family Medicine, College of Medicine, National Cheng Kung University, Tainan, Taiwan; 4 Department of Family Medicine, National Cheng Kung University Hospital, College of Medicine, National Cheng Kung University, Tainan, Taiwan; 5 Institute of Gerontology, College of Medicine, National Cheng Kung University, Tainan, Taiwan; 6 Division of Nephrology, Department of Internal Medicine, National Cheng Kung University Hospital, College of Medicine, National Cheng Kung University, Tainan, Taiwan; 7 Institute of Clinical Medicine, College of Medicine, National Cheng Kung University, Tainan, Taiwan; 8 Department of Pharmacy, College of Medicine, National Cheng Kung University, Tainan, Taiwan

## Abstract

**Importance:**

Increasing numbers of post hoc analyses have applied restricted mean survival time (RMST) analysis on the aggregated-level data from clinical trials to report treatment effects, but studies that use individual-level claims data are needed to determine the feasibility of RMST analysis for quantifying treatment effects among patients with type 2 diabetes in routine clinical settings.

**Objectives:**

To apply RMST analysis for assessing sodium-glucose cotransporter-2 inhibitor (SGLT2i)–associated cardiovascular (CV) events and estimating heterogenous treatment effects (HTEs) on CV and kidney outcomes in routine clinical settings.

**Design, Setting, and Participants:**

This comparative effectiveness study of Taiwan’s National Health Insurance Research Database examined 21 144 propensity score (PS)-matched pairs of patients with type 2 diabetes with SGLT2i and dipeptidyl peptidase-4 inhibitor (DPP4i) treatment for assessing CV outcomes, and 19 951 PS-matched pairs of patients with type 2 diabetes with SGLT2i and DPP4i treatment for assessing kidney outcomes. Patients were followed until December 31, 2018. Statistical analysis was performed from August 2021 to April 2022.

**Exposures:**

Newly stable SGLT2i or DPP4i use in 2017.

**Main Outcomes and Measures:**

Study outcomes were CV events including hospitalization for heart failure (HHF), 3-point major adverse CV events (3P-MACE: nonfatal myocardial infarction [MI], nonfatal stroke, and CV death), 4-point MACE (4P-MACE: HHF and 3P-MACE), and all-cause death, and chronic kidney disease (CKD). RMST and Cox modeling analyses were applied to estimate treatment effects on study outcomes.

**Results:**

After PS matching, the baseline patient characteristics were comparable between 21 144 patients with stable SGLT2i use (eg, mean [SD] age: 58.3 [10.7] years; 11 990 [56.7%] male) and 21 144 patients with stable DPP4i use (eg, mean [SD] age: 58.1 [11.6] years; 12 163 [57.5%] male) for assessing CV outcomes, and those were also comparable between 19 951 patients with stable SGLT2i use (eg, mean [SD] age: 58.1 [10.7] years; 11 231 [56.2%] male) and 19 951 patients with stable DPP4i use (eg, mean [SD] age: 57.9 [11.5] years; 11 340 [56.8%] male) for assessing kidney outcome. The 2-year difference in RMST between patients with SGLT2i use and patients with DPP4i use was 4.99 (95% CI, 3.56-6.42) days for HHF, 4.12 (95% CI, 2.72-5.52) days for 3P-MACE, 7.72 (95% CI, 5.83-9.61) days for 4P-MACE, 1.26 (95% CI, 0.47-2.04) days for MI, 2.70 (95% CI, 1.57-3.82) days for stroke, 0.69 (95% CI, 0.28-1.11) days for CV death, 6.05 (95% CI, 4.89-7.20) days for all-cause death, and 14.75 (95% CI, 12.99-16.52) days for CKD. Directions of hazard ratios from Cox modeling analyses were consistent with RMST estimates. No association was found between study treatment and the negative control outcome (dental visits for tooth care). Consistent results across sensitivity analyses using high-dimensional PS-matched and PS-weighting approaches supported the validity of primary analysis results. Largest difference in RMST of SGLT2i vs DPP4i use for HHF and CKD was found among patients with established heart failure (30.80 [95% CI, 5.08-56.51] days) and retinopathy (40.43 [95% CI, 31.74-49.13] days), respectively.

**Conclusions and Relevance:**

In this comparative effectiveness study, RMST analysis was feasible for translating treatment effects into more clinical intuitive estimates and valuable for quantifying HTEs among diverse patients in routine clinical settings.

## Key Points

**Question:**

Is restricted mean survival time (RMST) analysis feasible to translate comparative cardiovascular and kidney outcomes of sodium-glucose cotransporter-2 inhibitor (SGLT2i) vs dipeptidyl peptidase-4 inhibitor (DPP4i) therapy in routine clinical settings?

**Findings:**

In this comparative effectiveness study of 21 144 propensity score–matched pairs of patients with stable SGLT2i and DPP4i use for cardiovascular outcomes and 19 951 matched pairs for kidney outcomes, RMST analysis translated the SGLT2i-associated cardiorenal outcomes into clinical intuitive estimates, and estimated heterogenous treatment effects among patients with diverse characteristics in routine clinical settings.

**Meaning:**

This study’s findings suggest the feasibility of using RMST analysis to supplement traditional survival analyses for investigating cardiorenal outcomes among diverse patients with SGLT2i treatment.

## Teaser

This comparative effectiveness study applies restricted mean survival time (RMST) analysis for assessing sodium-glucose cotransporter-2 inhibitor SGLT-2 inhibitor (SGLT2i)–associated cardiovascular (CV) events and estimating heterogenous treatment effects (HTEs) on CV and kidney outcomes in routine clinical settings.

## Introduction

Restricted mean survival time (RMST) analysis is considered as a supplement to traditional Cox proportional hazards model (PHM) analysis^1,2^ in many fields (eg, cardiology,^2,3,4^ oncology,^5,6,7^ diabetes^8,9^). RMST refers to the mean survival time from an event over a specific (restricted) time horizon.^2,3,8,10^ The absolute difference in RMST between treatments provides an anchor for quantifying treatment effect without imposing any model assumptions.^1^ Unlike RMST analysis, Cox PHM analysis requires fulfilling the assumption of constant proportional hazards between comparison groups over time to provide valid hazard ratios (HRs) for quantifying treatment effect. In other words, the HR would be difficult to interpret when the constant hazard assumption may not be true. Also, considering that HRs are relative estimates and baseline hazards of the comparison or control group at each time point are not always explicitly provided, the absolute magnitude of hazards in the treatment group is unclear. In contrast, a difference in RMST using the RMST from the comparison or control group as a reference value can quantify treatment effect in a more clinical meaningful manner. Hence, RMST analysis provides more clinically interpretable estimates which can explicitly translate the treatment benefits into event-free time (eg, days) and further be applied to estimate health care savings and humanistic benefits (eg, quality of life) more intuitively.

RMST analysis was recently used for reporting treatment effect in endocrinology medicine,^8,9^ but they were post hoc analyses for cardiovascular outcomes and mortality trials in type 2 diabetes. In addition, due to lacking access to original trial records, previous studies reconstructed individual time-to-event data from published Kaplan-Meier (KM) curves to assess the RMST of treatment groups over the trial follow-up period.^2,8^ However, the validity of RMST estimates is of concern given the uncertainty regarding the consistency between the original and reconstructed data.^11^ Moreover, due to highly selective trial participants and a placebo (or usual care)–controlled trial design, the generalizability of trial findings to routine clinical settings, which comprise diverse patient populations and multiple competing treatments, is limited. Hence, the comparative effectiveness research using patient-level, claims data are warranted to assess the applicability of RMST analysis in routine clinical settings and affirm that difference in RMST can be used as a patient-centered measure of treatment benefit.

This study first determined the applicability of RMST analysis to estimate the treatment outcomes in routine clinical settings using well-known cardiovascular (CV) benefits associated with sodium-glucose cotransporter-2 inhibitors (SGLT2is) (eg, hospitalization for heart failure [HHF]) as an example. Then, given limited studies on SGLT2i-asscociated kidney outcomes, we further used RMST and Cox PHM analyses to estimate the SGLT2i-associated treatment outcomes in chronic kidney disease (CKD), and then explored the heterogeneous treatment effects (HTEs) on HHF and CKD among patient populations with diabetes in routine clinical settings.

## Methods

### Study Design and Data Source

This comparative effectiveness study used an active-comparator and new-user design^12^ based on Taiwan’s National Health Insurance Research Database (NHIRD). Briefly, Taiwan’s NHI program covers health care services for more than 99% of the Taiwanese population. The NHIRD comprises individual-level, encrypted, and deidentified health care records, including outpatient visits, emergency department visits, inpatient admissions, and prescription information.^13^ This study was approved by the National Cheng Kung University Hospital institutional review board and informed patient consent was waived because the study used deidentified patient data. This study followed the International Society for Pharmacoeconomics and Outcomes Research (ISPOR) reporting guideline.

### Cohort Identification

First, patients with type 2 diabetes with stable use of SGLT2is or dipeptidyl peptidase-4 inhibitors (DPP4is) were identified to avoid misclassification of study cohorts due to including short-term use of study drugs (eFigure 1 in Supplement 1). The first date of treatment initiation in 2017 was defined as the index date. Second, to include new users of study drugs, patients with exposure to either SGLT2is or DPP4is in the year prior to the index date were excluded. Third, patients aged less than 18 years at the index date or with death records before the index date by the Cause of Mortality data were excluded to avoid misclassification of study events. Fourth, considering that SGLT2i therapy is not recommended to patients with severe kidney impairment,^14^ patients who had any medical records of end-stage kidney disease or kidney transplantation before the index date by the Registry for Catastrophic Illness Patients were excluded. Lastly, patients with both SGLT2i and DPP4i use at the index date were excluded to ensure that SGLT2i and DPP4i groups were mutually exclusive. The study cohort for aim 1 (CV outcomes) was obtained using the aforementioned operational steps. Based on this cohort population, patients with any CKD diagnoses in the outpatient or inpatient files of the NHIRD within 1 year prior to the index date were excluded to obtain another study cohort for aim 2 (kidney outcome).

To enhance the between-group comparability and the control for confounding by indication, patients with stable SGLT2i use and patients with stable DPP4i use were 1:1 matched based on 5-to-1 digit propensity score (PS) greedy matching^15^ in the primary analyses. The PS for each study participant was estimated using a logistic regression model analysis to model SGLT2is vs DPP4is as a function of a series of baseline patient characteristics (Table 1).^16,17^ The operational definitions of baseline patient comorbidity characteristics are detailed in eTable 1 in Supplement 1. Of note, the PS matching (PSM) procedures were separately performed in aim 1 (CV outcomes) and aim 2 (kidney outcome) cohorts.

**Table 1.** Baseline Patient Characteristics of Study Cohorts After Propensity Score Matching

Characteristics	Cohort for CVD in aim 1		SMD^a^	Cohort for CKD in aim 2		SMD^a^
	DPP4is	SGLT2is		DPP4is	SGLT2is	
No. of patients	21 144	21 144		19 951	19 951	
Age at index date, mean (SD), y	58.1 (11.6)	58.3 (10.7)	0.01	57.9 (11.5)	58.1 (10.7)	0.01
Male, %	57.5	56.7	–0.02	56.8	56.2	–0.02
No. of GLDs in year before index date, mean (SD)	1.6 (1.0)	1.5 (1.0)	–0.01	1.5 (1.0)	1.5 (1.0)	–0.01
Duration of diabetes at index date, mean (SD), y	8.3 (3.2)	8.3 (3.1)	0.01	8.2 (3.2)	8.3 (3.2)	0.01
Surrogate indicators for baseline kidney function, %						
Participants in pre-ESRD program in year before index date	1.1	1.0	–0.04	0.4	0.1	–0.05
Metformin prescribed within 90 d before index date	40.3	40.3	0.00	40.9	40.6	0.00
Acarbose prescribed within 90 d before index date	10.7	10.6	–0.00	10.8	10.6	–0.00
Previous health care service utilization in year before index date						
No. of glycated hemoglobin test, mean (SD)	6.0 (3.0)	6.0 (2.8)	0.03	5.9 (3.0)	5.9 (2.7)	0.00
No. of low-density lipoprotein tests, mean (SD)	4.0 (2.6)	4.0 (2.6)	0.00	3.9 (2.5)	3.9 (2.5)	0.00
Patients who took bone mineral density tests, %	0.4	1.1	0.01	0.9	1.0	0.01
Diabetes-related complications in year before index date, %						
Chronic kidney disease	5.5	5.5	–0.00	NA	NA	NA
Neuropathy	8.9	9.1	0.01	8.5	9.0	0.01
Retinopathy	7.7	7.7	0.00	7.9	7.4	0.00
Peripheral vascular disease	3.5	3.3	–0.01	3.3	3.1	–0.01
Cerebrovascular disease	4.4	4.4	0.00	4.4	4.3	0.00
Cardiovascular disease	19.0	19.2	0.00	18.8	18.9	0.00
Heart failure	3.3	3.4	0.00	3.1	3.2	0.00
Acute myocardial infarction	1.6	1.7	0.01	1.6	1.7	0.01
Ischemic heart disease	12.5	12.6	0.00	12.6	12.4	0.00
Diabetic ketoacidosis	0.1	0.1	–0.01	0.1	0.1	–0.01
Hypoglycemia	0.2	0.3	0.00	0.2	0.2	0.00
GLDs prescribed in year before index date, %						
Metformin	55.4	54.4	–0.02	55.5	54.6	–0.02
Sulfonylureas	46.8	46.0	–0.02	46.3	45.9	–0.02
Meglitinides	5.6	5.5	–0.00	5.5	5.4	–0.00
Thiazolidinediones	16.6	16.7	0.00	16.4	16.7	0.00
Acarbose	16.9	17.2	0.01	16.8	17.2	0.01
GLP1-RAs	0.7	0.9	0.02	0.7	0.9	0.02
Insulins	17.5	18.2	0.02	16.8	17.5	0.02
CVD-related medication history in year before index date, %						
Lipid-lowering medications	78.8	78.8	–0.00	78.2	78.3	–0.00
Alpha blockers	4.3	4.2	–0.01	3.8	3.9	–0.01
Beta blockers	33.8	33.5	–0.01	33.1	33.1	–0.01
RAAS agents	61.4	61.5	0.00	60.9	60.6	0.00
Diuretics	13.6	13.6	–0.00	12.8	13.0	–0.00
Calcium channel blockers	28.8	28.5	–0.01	28.2	28.2	–0.01
Anti-arrhythmics	2.3	2.2	–0.01	2.3	2.1	–0.01
Cardiac glycosides	1.2	1.2	–0.00	1.2	1.1	–0.00
Vasodilators	12.6	12.7	0.00	12.4	12.4	0.00
Antiplatelets	35.1	35.1	0.00	34.4	34.5	0.00
Anticoagulants	1.7	1.9	0.01	1.7	1.8	0.01

Abbreviations: CKD, chronic kidney disease; CVD, cardiovascular disease; DPP4is, dipeptidyl peptidase-4 inhibitors; ESRD, end-stage renal disease; GLDs, glucose-lowering drugs; GLP1-RAs, glucagon-like peptide 1-receptor agonists; NA, not applicable; RAAS, renin-angiotensin-aldosterone system; SGLT2is, sodium-glucose cotransporter-2 inhibitors; SMD, standardized mean difference.

^a^ An absolute value of SMD greater than 0.1 indicates a statistically significant difference in patient characteristics between drug groups. Notably, a greater between-group comparability in these baseline characteristics was achieved as all SMDs are less than 0.1.

### Drug Exposure and Study Outcome Assessment

The Anatomical Therapeutic Chemical Classification System from the World Health Organization was applied to identify drug exposure. The study outcomes for aim 1^13^ were CV events including HHF, 3-point major adverse cardiovascular events (3P-MACE: nonfatal stroke, nonfatal myocardial infarction [MI], or CV death), 4P-MACE (HHF or 3P-MACE), nonfatal stroke, nonfatal MI, CV death, and all-cause death, and the study outcome for aim 2 was CKD (eTable 2 in Supplement 1). Mortality status was ascertained using the Cause of Mortality data in the NHIRD. Patients were followed from the index date until the occurrence of a study outcome, loss of follow-up, or December 31, 2018, whichever came first.

### Statistical Analyses

The standardized mean difference (SMD) was used to determine the balance in baseline patient characteristics between treatment groups before and after the matching, with an absolute value of SMD less than 0.1 indicating an insignificant between-group difference. Primary analyses included RMST and Cox PHM analyses on each study outcome. Briefly, the KM survival curves of study outcomes for treatment groups over a specific time interval were first plotted based on individual-level time-to-event data and the RMST of each drug group was then estimated based on the area under the KM curve.^18^ The time horizon for a given outcome was determined as the minimum of the largest followed-up time of patients with SGLT2i use and patients with DPP4i use. The difference in RMST between the SGLT2i and DPP4i groups with the associated 95% CI was finally estimated for each study outcome. The hazard ratios (HRs) and associated 95% CIs with SGLT2i vs DPP4i use for each study outcome from the Cox model analyses were also estimated for comparison with the estimates of difference in RMST in terms of the direction of findings. Statistical significance was determined if 95% CI for difference in RMST did not overlap with 0 or if 95% CI for HRs did not overlap with 1.

Several sensitivity analyses using a negative control outcome (ie, dental visits for tooth care),^19,20^ high-dimension propensity score–matching,^21,22,23,24^ and PS-weighting^25,26^ were performed to explore and diminish the effect of potential confounders that arise from the use of claims data in the estimation of RMST and HRs. A series of subgroup analyses based on baseline patient characteristics were performed to evaluate SGLT2i-associated HTEs for HHF and CKD. Sensitivity and subgroup analyses are detailed in eDescription 1 in Supplement 1. All analyses were performed from August 2021 to April 2022 and using SAS software version 9.4 (SAS Institute).

## Results

There were 21 144 PS-matched pairs of patients with stable use of SGLT2i and patients with stable use of DPP4i included in the study cohort for aim 1 (CV outcomes) and 19 951 PS-matched pairs included for aim 2 (kidney outcome) (eFigure 1 in Supplement 1). As shown in Table 1, after PSM, the baseline patient characteristics achieved satisfactory between-group balance among patients with SGLT2i use (eg, mean [SD] age: 58.3 [10.7] years; 11 990 [56.7%] male; mean [SD] diabetes duration: 8.3 [3.1] years; 4059 [19.2%] with established CV diseases) and patients with DPP4i use (eg, mean [SD] age: 58.1 [11.6] years; 12 163 [57.5%] male; mean [SD] diabetes duration: 8.3 [3.2] years; 4036 [19.0%] with established CV diseases) for assessing CV outcomes, and those were also comparable between patients with SGLT2i use (eg, mean [SD] age: 58.1 [10.7] years; 11 231 [56.2%] male; mean [SD] diabetes duration: 8.3 [3.2] years; 3784 [18.9%] with established CV diseases) and patients with DPP4i use (eg, mean [SD] age: 57.9 [11.5] years; 11 340 [56.8%] male; mean [SD] diabetes duration: 8.2 [3.2] years; 3759 [18.8%] with established CV diseases) for assessing the kidney outcome. eTable 3 in Supplement 1 provides patient baseline characteristics before the matching. The detailed PS distribution is shown in eFigure 2 in Supplement 1.

### Primary Analyses of CV Outcomes (Aim 1) and Kidney Outcome (Aim 2)

Table 2 indicates that over 2 years, the RMSTs of patients with SGLT2i use and patients with DPP4i use for CV outcomes ranged from 713.18 (95% CI, 711.9-714.4) days (for 4P-MACE) to 728.25 (95% CI, 728.0-728.5) days (for CV death) and from 705.35 (95% CI, 703.8-706.8) days (for 4P-MACE) to 727.55 (95% CI, 727.2-727.8) days (for CV death), respectively; those of patients with SGLT2i use and CKD was 720.48 (95% CI, 719.5-721.3) days and patients with DPP4i use and CKD was 705.52 (95% CI, 703.9-707.0) days. Accordingly, the use of SGLT2is vs DPP4is delayed the mean time from the occurrence of HHF by 4.99 (95% CI, 3.56 to 6.42) days, 3P-MACE by 4.12 (95% CI, 2.72-5.52) days, 4P-MACE by 7.72 (95% CI, 5.83-9.61) days, MI by 1.26 (95% CI, 0.47-2.04) days, stroke by 2.70 (95% CI, 1.57-3.82) days, CV death by 0.69 (95% CI, 0.28-1.11) days, all-cause death by 6.05 (95% CI, 4.89-7.20) days, and CKD by 14.75 (95% CI, 12.99-16.52) days. Additionally, the HR of SGLT2is vs DPP4is using Cox model analyses was 0.61 (95% CI, 0.53 to 0.70) for HHF, 0.70 (0.61-0.79) for 3P-MACE, 0.67 (0.60-0.73) for 4P-MACE, 0.71 (0.57-0.90) for MI, 0.69 (0.59-0.80) for stroke, 0.51 (0.35-0.76) for CV death, 0.46 (0.39-0.53) for all-cause death, and 0.38 (0.33-0.43) for CKD. Details of survival curves of the SGLT2i and DPP4i groups for each outcome are given in eFigure 3 in Supplement 1.

**Table 2.** Restricted Mean Survival Time and Cox Proportional Hazards Model Analyses on Study Outcomes Among Propensity Score–Matched SGLT2i and DPP4i Groups (Primary Analyses)

	Estimated event rate (events/100 person years)		RMST (95% CI), d^a^			Estimated HR associated with SGLT2i vs DPP4i use (95% CI)
	SGLT2is	DPP4is	SGLT2is	DPP4is	Difference	
Cardiovascular outcomes in aim 1						
HHF	1.05	1.72	720.71 (719.8-721.6)	715.65 (714.5-716.7)	4.99 (3.5-6.4)	0.61 (0.53-0.70)
3P-MACE^b^	1.26	1.81	719.96 (719.0-720.8)	715.78 (714.6-716.8)	4.12 (2.7-5.5)	0.70 (0.61-0.79)
4P-MACE^c^	2.10	3.15	713.18 (711.9-714.4)	705.35 (703.8-706.8)	7.72 (5.8-9.6)	0.67 (0.60-0.73)
Myocardial infarction	0.38	0.54	726.23 (725.7-726.7)	724.95 (724.3-725.5)	1.26 (0.4-2.0)	0.71 (0.57-0.90)
Stroke	0.80	1.16	723.20 (722.4-723.9)	720.47 (719.5-721.3)	2.70 (1.5-3.8)	0.69 (0.59-0.80)
Cardiovascular death	0.12	0.23	728.25 (728.0-728.5)	727.55 (727.2-727.8)	0.69 (0.2-1.1)	0.51 (0.35-0.76)
All-cause death	0.76	1.66	723.85 (723.1-724.5)	717.72 (716.7-718.6)	6.05 (4.8-7.2)	0.46 (0.39-0.53)
Kidney outcome in aim 2						
CKD	1.16	3.07	720.48 (719.5-721.3)	705.52 (703.9-707.0)	14.75 (12.9-16.5)	0.38 (0.33-0.43)

Abbreviations: CKD, chronic kidney disease; DPP4is, dipeptidyl peptidase-4 inhibitors; HHF, hospitalization for heart failure; HR, hazard ratio; RMST, restricted mean survival time; SGLT2is, sodium-glucose cotransporter-2 inhibitors; 3P-MACE, 3-point major adverse cardiovascular event; 4P-MACE, 4-point major adverse cardiovascular event.

^a^ 729 days (1.99 years) was the minimum of the largest observed event time in study drug groups and specified in the restricted mean survival time analysis.

^b^ 3P-MACE comprised nonfatal myocardial infarction, nonfatal stroke, and cardiovascular death.

^c^ 4P-MACE comprised nonfatal hospitalized heart failure, nonfatal myocardial infarction, nonfatal stroke, and cardiovascular death.

### Sensitivity Analyses: Negative Control Outcome, High-Dimensional PS-Matched and PS-Weighted Cohorts

No statistical association between SGLT2i vs DPP4i use and tooth care was found (ie, difference in RMST of 0.09 days [95% CI, –0.19 to 0.38 days] and HR of 0.78 [0.41-1.47] were found for SGLT2i vs DPP4i use over 2 years). The magnitudes of postponement of study outcomes from using SGLT2is vs DPP4is and associated HRs in the sensitivity analyses based on high-dimensional PS-matched cohorts (Table 3) are in line with primary analysis findings based on PS-matched cohorts (Table 2). HHF outcomes were postponed by an RMST of 3.77 (95% CI, 2.36 to 5.18) days, 3P-MACE outcomes postponed by 2.55 (95% CI, 1.20-3.91) days, 4P-MACE outcomes postponed by 5.13 (95% CI, 3.29-6.97) days, MI outcomes postponed by 0.89 (95% CI, 0.15-1.64) days, stroke outcomes postponed by 1.45 (95% CI, 0.36-2.55) days, CV death postponed by 0.56 (95% CI, 0.14-0.97), all-cause death postponed by 3.56 (95% CI, 2.48-4.63), and CKD outcomes by 15.46 (95% CI, 13.58-17.34) days, by using SGLT2is vs DPP4is. In addition, the estimated HRs derived from 3 PS-weighted pseudocohorts (ie, IPTW, stabilized IPTW, SMRW) for CV and kidney outcomes (eTable 4 in Supplement 1) were comparable with the results from the analyses of PS-matched cohorts (Table 2).

**Table 3.** RMST and Cox Proportional Hazards Model Analyses on Negative Control Outcome Among Propensity-Score Matched SGLT2i and DPP4i Groups and Study Outcomes Among High-Dimensional Propensity-Score-Matched SGLT2i and DPP4i Groups (Sensitivity Analyses)

	Estimated event rate (events/100 person-years)		RMST (95% CI), d^a^			Estimated HR associated with SGLT2i vs DPP4i use (95% CI)
	SGLT2is	DPP4is	SGLT2is	DPP4is	Difference	
Negative control outcome						
Dental visits for tooth care	0.05	0.07	728.61 (728.4 to 728.8)	728.51 (728.3 to 728.7)	0.09 (–0.1 to 0.3)	0.78 (0.41 to 1.47)
Cardiovascular outcomes in aim 1						
HHF	1.05	1.56	720.68 (719.7 to 721.6)	716.86 (715.7 to 717.9)	3.77 (2.3 to 5.1)	0.76 (0.60 to 0.96)
3P-MACE^b^	1.25	1.61	720.00 (719.0 to 720.9)	717.41 (716.3 to 718.4)	2.55 (1.2 to 3.9)	0.78 (0.68 to 0.88)
4P-MACE^c^	2.10	2.82	713.18 (711.9 to 714.4)	707.97 (706.5 to 709.3)	5.13 (3.2 to 6.9)	0.74 (0.67 to 0.82)
Myocardial infarction	0.36	0.48	726.39 (725.9 to 726.8)	725.48 (724.9 to 726.0)	0.89 (0.1 to 1.6)	0.80 (0.68 to 0.94)
Stroke	0.81	1.02	723.10 (722.3 to 723.8)	721.63 (720.8 to 722.4)	1.45 (0.3 to 2.5)	0.67 (0.58 to 0.77)
Cardiovascular death	0.12	0.20	728.23 (727.9 to 728.4)	727.67 (727.3 to 728.0)	0.56 (0.1 to 0.9)	0.59 (0.39 to 0.88)
All-cause death	0.77	1.32	723.78 (723.1 to 724.4)	720.17 (719.3 to 721.0)	3.56 (2.4 to 4.6)	0.58 (0.50 to 0.68)
Kidney outcome in aim 2						
CKD	1.21	3.18	720.09 (719.1 to 721.0)	704.41 (702.7 to 706.0)	15.46 (13.5 to 17.3)	0.38 (0.34 to 0.43)

Abbreviations: CKD, chronic kidney disease; DPP4is, dipeptidyl peptidase-4 inhibitors; HHF, hospitalization for heart failure; HR, hazard ratio; RMST, restricted mean survival time; SGLT2is, sodium-glucose cotransporter-2 inhibitors; 3P-MACE, 3-point major adverse cardiovascular event; 4P-MACE, 4-point major adverse cardiovascular event.

^a^ 729 days (1.99 years) was the minimum of the largest observed event time in study drug groups and specified in the restricted mean survival time analysis.

^b^ 3P-MACE comprised nonfatal myocardial infarction, nonfatal stroke, and cardiovascular death.

^c^ 4P-MACE comprised hospitalization for heart failure, nonfatal myocardial infarction, nonfatal stroke, and cardiovascular death.

### Subgroup Analyses for HHF in Aim 1 and CKD in Aim 2

**Figure 1.**
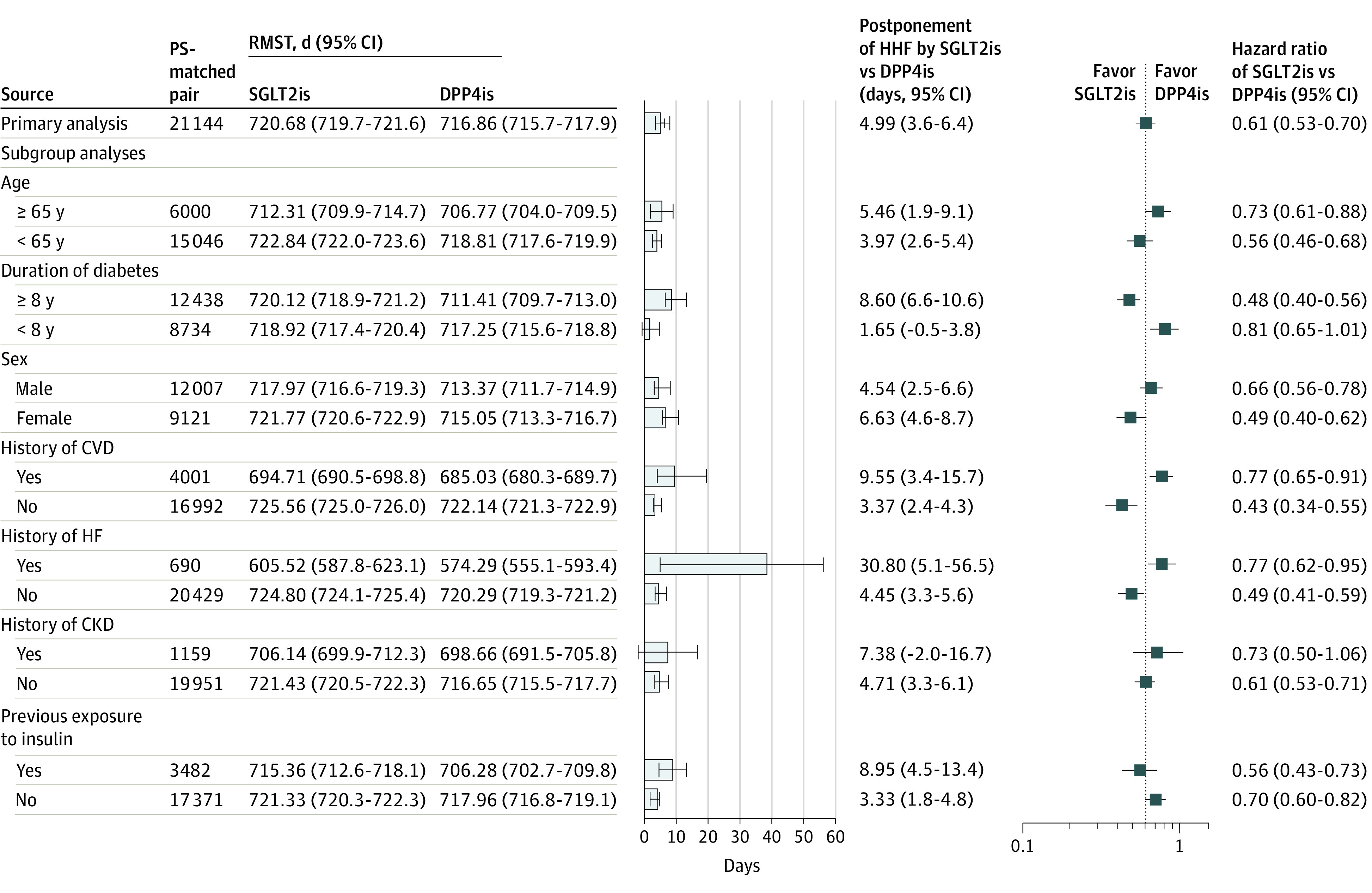
Subgroup Analyses of Hospitalization for HHF Associated With SGLT2i vs DPP4i Use CKD indicates chronic kidney disease; CVD, cardiovascular disease; DPP4i, dipeptidyl peptidase-4 inhibitor; HF, heart failure; HHF, history of heart failure; PS, propensity score; RMST, restricted mean survival time; SGLT2i, sodium-glucose cotransporter-2 inhibitor.

**Figure 2.**
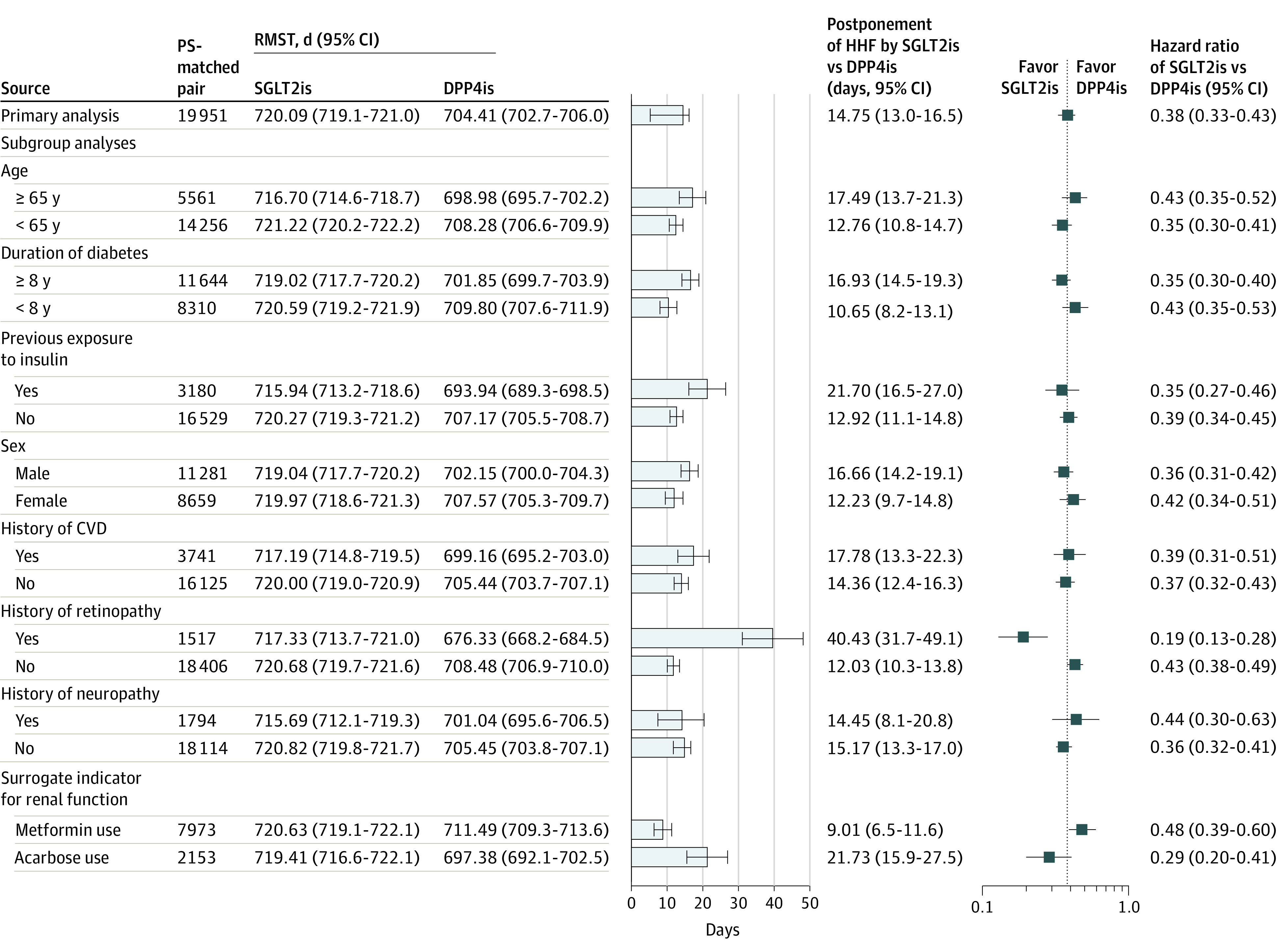
Subgroup Analyses of CKD Associated With SGLT2i vs DPP4i Use CKD indicates chronic kidney disease; CVD, cardiovascular disease; DPP4i, dipeptidyl peptidase-4 inhibitor; PS, propensity score; RMST, restricted mean survival time; SGLT2i, sodium-glucose cotransporter-2 inhibitor.

All subgroup analyses on the HHF outcome show significantly favorable results (in terms of difference in RMST and estimated HRs) for the use of SGLT2is vs DPP4is, except for the analyses among patients with a diabetes duration of shorter than 8 years and those with CKD history. However, treatment heterogeneity of SGLT2i vs DPP4i use on HHF across these subgroups was found, given a wide range of estimates of difference in RMST from 1.65 (95% CI, –0.52 to 3.82) days for patients with a diabetes duration of shorter than 8 years to 30.80 (95% CI, 5.08-56.51) days for those with established HF events (Figure 1). Treatment heterogeneity on CKD outcome across patient subgroups was also found, with the smallest between-treatment group difference in RMST among patients receiving metformin (9.01 [95% CI, 6.47-11.55] days) and the largest RMST difference in the patient group with a history of retinopathy (40.43 [31.74-49.13] days) (Figure 2). Detailed event rates in patient subgroups are shown in eTables 5 and 6 in Supplement 1.

## Discussion

To our knowledge, this study is the first to support the applicability of RMST analyses in a routine clinical setting. Several rigorous methodologies were applied to overcome potential confounding and biases that arise with the use of claims data and a series of sensitivity analyses were conducted to confirm study robustness. Furthermore, HTEs in diverse patients under clinical practice were reported to facilitate individualized medicine. RMST estimates are both informative and interpretable for patients regarding their expectation of treatment benefit at its initiation, and thereby support their decisions to undertake and even adhere to treatment.^8^ Additionally, because RMST analyses translate the treatment-related survival benefit into a more clinically meaningful measure (eg, event-free time), corresponding health care savings and humanistic benefits (eg, quality of life) can be intuitively estimated.^27,28,29^ Therefore, RMST results could be an alternative metric for demonstrating the value of treatment in health technology assessment or reassessment, especially when the violation of proportional hazards is of concern,^30^ to support health care policy decisions and resource allocation.

### Applicability of RMST in Routine Clinical Settings

Previous trials reported that compared with placebo or sulfonylurea, SGLT2i therapy delayed the occurrence of 3P-MACE by 4.1 to 32 days.^8^ This study using claims data found that the use of SGLT2is vs DPP4is postponed CV events by 0.69 to 7.72 days (Table 2). Although direct comparison between clinical trials and studies using claims data should be done with caution due to the considerable differences in the study design and population (eg, inclusion and exclusion criteria, study follow-up, comparison groups), the SGLT2i-associated CV benefits indicated by RMST estimates are consistent between the present analysis using claims data and previous trials. This supports our study validity and supports the applicability of RMST analyses in routine clinical settings. Therefore, it is important to highlight the methodologies applied in this study to ensure the validity of RMST analyses in the assessment of treatment outcomes in routine clinical settings, including the PSM procedures used to achieve a greater level of between-group comparability,^25^ the negative control outcome analysis to corroborate the validity of study data and analytic procedures,^20^ high-dimensional PS techniques for eliminating potential unmeasured confounding in studies using claims data, which may provide more conservative estimates,^21,22,23,24^ and PS weighting methods (ie, IPTW, stabilized IPTW), which retained most of the original study cohort, to support the external validity of our findings.^25,26^ These rigorous methodologies allow RMST analyses to be conducted in routine clinical settings.

Difference in RMST can facilitate the making of intuitive inferences regarding relative treatment effects across different clinical outcomes and patient subgroups.^7^ In contrast, based on HRs alone, comparative treatment effects across different outcomes might not be explicit because baseline hazards for the control group could be different for different outcomes.^7,31^ Taking HHF and CV death outcomes as examples, although the use of SGLT2is vs DPP4is was associated with 39% and 49% decreases in the risk of HHF and CV death (HRs, 0.61 and 0.51), respectively, inference from a direct comparison of these estimates should be very done with caution because the association of DPP4is with the 2 outcomes is apparently different (eFigure 3 in Supplement 1). In contrast, the effect of the comparator group is ascertained in RMST analyses, which further facilitates the estimation of difference in RMST. Such absolute values can be applied to compare treatment effects across different outcomes. According to the estimates of difference in RMST, the absolute delay times from the occurrence of HHF and CV death while using SGLT2is vs DPP4is were 4.99 and 0.69 days, respectively. Based on such intuitive values, one could conclude that SGLT2i therapy yielded a more HHF-event-free benefit compared to its survival benefits from CV death. Further details of comparison and interpretation of RMST and HR estimates are provided in eDescription 2 in Supplement 1.

The utility of RMST analyses in this study using claims data was also demonstrated by the quantification of HTEs into a clinically meaningful measure.^32^ That is, the use of SGLT2is vs DPP4is could delay HHF occurrence by the shortest delay of 1.65 days in patients with diabetes duration less than 8 years to the longest delay of 30.80 days among those with established HF (Figure 1). Hence, in studies of treatment effects under clinical practice, RMST analyses should be used to supplement traditional survival analyses using Cox modeling. That is, RMST estimates reveal the magnitude of the treatment effect for individual treatments and the comparative effects of different treatment groups, and across different outcomes and diverse patient subgroups in routine clinical settings. Therefore, RMST estimates together with HRs could optimize clinical communication and treatment decisions in routine clinical settings.

### Utility of RMST for SGLT2i-Associated Kidney Benefit and HTEs in Routine Clinical Settings

Given limited studies on the association of SGLT2i therapy and the prevention of CKD among patients with diabetes, the HR and RMST estimates in this study add supporting evidence for the SGLT2i-associated benefit for the prevention of incident CKD under clinical practice. Specifically, the use of SGLT2is vs DPP4is was associated with a 62% decreased risk for CKD (HRs of 0.38 [95% CI, 0.33-0.43] and 0.38 [95% CI, 0.34-0.43] for PSM-matched and high-dimensional PS-matched cohorts, respectively), which fell within the range of previously reported HR estimates (ie, 0.29 [95% CI, 0.22 to 0.38]^33^ to 0.44 [95% CI, 0.28-0.69]^34^). Additionally, the estimated difference in RMST in delaying CKD while using SGLT2is vs DPP4is over 2 years was 14.75 (95% CI, 12.99-16.52) days (RMST for patients with stable SGLT2i use: 720.48 [95% CI, 719.56-721.39] days; RMST for patients with stable DPP4i use: 705.52 [95% CI, 703.98-707.06] days); these are informative and intuitive values for physicians and patients (ie, they indicate that SGLT2i therapy could extend the event-free time of CKD by nearly half a month over 2 years). Moreover, our subgroup analysis results (Figure 2) support kidney benefits with SGLT2i therapy across diverse patient populations in routine clinical settings. Patients who have diabetes with retinopathy may benefit most from SGLT2is (ie, using SGLT2is vs DPP4is among patients who have diabetes with retinopathy could delay the occurrence of CKD by approximately 40 days. Diabetic retinopathy may be representative of systemic microvascular damage secondary to diabetes and is associated with composite kidney end points.^35,36^ This implies that among patients with diabetes and retinopathy in routine clinical settings who are known to be at an increased risk for developing CKD,^37,38^ timely intervention with SGLT2i therapy may maximize treatment benefit in terms of long-term kidney outcomes of patients.

### Limitations

Several limitations of this study should be acknowledged. First, patient laboratory records (eg, HbA_1c_) reflecting disease severity were not available in our study administrative data. This may affect the validity of outcome assessment. To minimize this concern, a large number of measurable indicators were considered in the PSM procedure and the unmeasurable confounder issue was addressed using advanced hdPS techniques. Second, because of the limited study period (ie, 2 years), the postponements of the occurrence of CV and kidney events following SGLT2i vs DPP4i initiation (presented in days) were relatively small (ie, few days to 1 month). Additionally, inference based on our RMST results was limited to 2 years, and the extrapolation of RMST estimates beyond this prespecific time was prohibited. Despite the limited study period, estimates of difference in RMST were still statistically significant across all analyses. Therefore, one may expect that difference in RMST could be magnified with a longer study follow-up period. Lastly, because the RMST analyses were based on KM curves, our RMST estimates might experience problems commonly seen with KM curves (eg, competing risk, noninformative censoring).

## Conclusion

Along with the application of rigorous methodologies to minimize potential confounding and biases that arise with the use of claims data, this study adds supporting clinical research evidence for the feasibility of using RMST analyses as a supplement to traditional survival analyses for investigating treatment effects in routine clinical settings. Our assessments of comparative CV and kidney outcomes of SGLT2i vs DPP4i therapy provide translated and intuitive evidence that can facilitate patient-clinician communication to optimize shared decision-makings and quantify HTEs among diverse patient populations to promote individualized medicine in routine clinical settings.

## Notes

Supplement 1. **eDescription 1.** Sensitivity and Subgroup Analyses **eTable 1.** Strengthening the Reporting of Observational Studies in Epidemiology Checklist **eTable 2.** Operational Definitions of Baseline Characteristics Defined by ICD-9-CM and ICD-10-CM Disease Diagnosis Codes **eTable 3.** Operational Definitions of Study Outcomes Defined by ICD-9-CM and ICD-10-CM Disease Diagnosis Codes and Data Sources for Measurement **eTable 4.** Baseline Characteristics of Study Cohorts Before Propensity Score Matching **eTable 5.** Results of Cox Proportional Hazard Model Analyses on Study Outcomes Among Cohorts Using Either High-Dimensional Propensity-Score or Propensity-Score Weighting Procedures (Sensitivity Analyses) **eTable 6.** Event Rate and Hazard Ratio Associated With SGLT2i Versus DPP4i Use in Hospitalization for Heart Failure Outcome (Subgroup Analyses) **eTable 7.** Event Rate and Hazard Ratio Associated With SGLT2i Versus DPP4i Use in Chronic Kidney Disease Outcome (Subgroup Analyses) **eFigure 1.** Flowchart of Study Cohort Selection **eFigure 2.** Kernel Density Plots of Propensity Score for Study Cohorts (A) Before and (B) After Propensity Score Matching for Aim 1 and Those for Study Cohort (C) Before and (D) After Propensity Score Matching for Aim 2 **eFigure 3.** Kaplan-Meier Survival Curves of SGLT2i and DPP4i Users for (A) Hospitalization for Heart Failure, (B) 3P-MACE, (C) 4P-MACE, (D) Myocardial Infarction, (E) Stroke, (F) Cardiovascular Death, (G) All-Cause Death, (H) Chronic Kidney Disease, and (I) Dental Visits for Tooth Care **eDescription 2.** Comparison and Interpretations of Restricted Mean Survival Time Analysis and Hazards Ratio Estimates

Supplement 2.
Data Sharing Statement
