# Errors in End Matter and Supplement 1

**DOI:** 10.1001/jamanetworkopen.2022.54512

**Published:** 2023-01-24

In the Original Investigation titled “Restricted Mean Survival Time Analysis to Estimate SGLT2i–Associated Heterogeneous Treatment Effects on Primary and Secondary Prevention of Cardiorenal Outcomes in Patients With Type 2 Diabetes in Taiwan,”^1^ published December 15, 2022, there were errors in the end matter and Supplement 1. In the end matter’s Supplement 1 table of contents and in Supplement 1, “eTable 1. Strengthening the Reporting of Observational Studies in Epidemiology Checklist” should have been deleted and all eTables renumbered accordingly. This article has been corrected.^1^
