# Supplementary material for: Restricted Mean Survival Time Analysis to Estimate SGLT2i–Associated Heterogeneous Treatment Effects on Primary and Secondary Prevention of Cardiorenal Outcomes in Patients With Type 2 Diabetes in Taiwan
Source: JAMA Netw Open. 2022 Dec 15;5(12):e2246928. doi: 10.1001/jamanetworkopen.2022.46928 (PMC9856417; doi:10.1001/jamanetworkopen.2022.46928)
Supplement: Supplement 1. — eDescription 1. Sensitivity and Subgroup Analyses eTable 1. Strengthening the Reporting of Observational Studies in Epidemiology Checklist eTable 2. Operational Definitions of Baseline Characteristics Defined by ICD-9-CM and ICD-10-CM Disease Diagnosis Codes eTable 3. Operational Definitions of Study Outcomes Defined by ICD-9-CM and ICD-10-CM Disease Diagnosis Codes and Data Sources for Measurement eTable 4. Baseline Characteristics of Study Cohorts Before Propensity Score Matching eTable 5. Results of Cox Proportional Hazard Model Analyses on Study Outcomes Among Cohorts Using Either High-Dimensional Propensity-Score or Propensity-Score Weighting Procedures (Sensitivity Analyses) eTable 6. Event Rate and Hazard Ratio Associated With SGLT2i Versus DPP4i Use in Hospitalization for Heart Failure Outcome (Subgroup Analyses) eTable 7. Event Rate and Hazard Ratio Associated With SGLT2i Versus DPP4i Use in Chronic Kidney Disease Outcome (Subgroup Analyses) eFigure 1. Flowchart of Study Cohort Selection eFigure 2. Kernel Density Plots of Propensity Score for Study Cohorts (A) Before and (B) After Propensity Score Matching for Aim 1 and Those for Study Cohort (C) Before and (D) After Propensity Score Matching for Aim 2 eFigure 3. Kaplan-Meier Survival Curves of SGLT2i and DPP4i Users for (A) Hospitalization for Heart Failure, (B) 3P-MACE, (C) 4P-MACE, (D) Myocardial Infarction, (E) Stroke, (F) Cardiovascular Death, (G) All-Cause Death, (H) Chronic Kidney Disease, and (I) Dental Visits for Tooth Care eDescription 2. Comparison and Interpretations of Restricted Mean Survival Time Analysis and Hazards Ratio Estimates [file jamanetwopen-e2246928-s001.pdf]

Supplemental Online Content

Peng ZY, Yang CT, Kuo S, Wu CH, Lin WH, Ou HT. Restricted mean survival time analysis to estimate SGLT2i–associated heterogeneous treatment effects on primary and secondary prevention of cardiorenal outcomes in patients with type 2 diabetes in Taiwan. *JAMA Netw Open.* 2022;5(12):e2246928. doi:10.1001/jamanetworkopen.2022.46928

eDescription 1. Sensitivity and Subgroup Analyses

eTable 1. Strengthening the Reporting of Observational Studies in Epidemiology Checklist

eTable 2. Operational Definitions of Baseline Characteristics Defined by ICD-9-CM and ICD-10-CM Disease Diagnosis Codes

eTable 3. Operational Definitions of Study Outcomes Defined by ICD-9-CM and ICD-10-CM Disease Diagnosis Codes and Data Sources for Measurement

eTable 4. Baseline Characteristics of Study Cohorts Before Propensity Score Matching

eTable 5. Results of Cox Proportional Hazard Model Analyses on Study Outcomes Among Cohorts Using Either High-Dimensional Propensity-Score or Propensity-Score Weighting Procedures (Sensitivity Analyses)

eTable 6. Event Rate and Hazard Ratio Associated With SGLT2i Versus DPP4i Use in Hospitalization for Heart Failure Outcome (Subgroup Analyses)

eTable 7. Event Rate and Hazard Ratio Associated With SGLT2i Versus DPP4i Use in Chronic Kidney Disease Outcome (Subgroup Analyses)

eFigure 1. Flowchart of Study Cohort Selection

eFigure 2. Kernel Density Plots of Propensity Score for Study Cohorts (A) Before and (B) After Propensity Score Matching for Aim 1 and Those for Study Cohort (C) Before and (D) After Propensity Score Matching for Aim 2

eFigure 3. Kaplan-Meier Survival Curves of SGLT2i and DPP4i Users for (A) Hospitalization for Heart Failure, (B) 3P-MACE, (C) 4P-MACE, (D) Myocardial Infarction, (E) Stroke, (F) Cardiovascular Death, (G) All-Cause Death, (H) Chronic Kidney Disease, and (I) Dental Visits for Tooth Care

© 2022 Peng ZY et al. *JAMA Network Open.*

eDescription 2. Comparison and Interpretations of Restricted Mean Survival Time Analysis and Hazards Ratio Estimates

This supplemental material has been provided by the authors to give readers additional information about their work.

© 2022 Peng ZY et al. *JAMA Network Open.*

eDescription 1. Sensitivity and subgroup analyses

Sensitivity analyses

First, a negative control outcome analysis was applied to strengthen causal inference regarding drug exposure and outcomes of interest. Theoretically, study outcomes of interest (e.g., hospitalization for heart failure [HHF], chronic kidney disease [CKD]) have the same potential sources of bias as those for a negative control outcome that is not plausibly related to the drug exposure of interest (i.e., SGLT2is or DPP4is). We identified dental visits for tooth care as a negative control given that there is no evidence of an association between the study drugs and tooth care. Second, instead of using the propensity-score matching (PSM) approach, the high-dimension propensity score (hdPS) approach (i.e., prespecified variables in the PSM together with 400 covariates that were automatically derived and prioritized from a total of 1,000 empirical variables in five data dimensions), which can mitigate potentially unmeasurable confounding effects in pharmacoepidemiology research using administrative data, was applied to re-match SGLT2i and DPP4i users. The primary analyses were repeated based on the hdPS-matched cohort. Lastly, the estimated HRs of study outcomes based on the PS-weighted pseudo-cohorts derived from three PS-weighting techniques, namely inverse probability of treatment weighting (IPTW), stabilized IPTW, and standardized mortality ratio weighting (SMRW), were measured and compared with those from the PS-matched and hdPS-matched cohorts.

Subgroup analyses

For HHF, the analyses were conducted in subgroups by age, gender, diabetes duration, histories of cardiovascular diseases, heart failure (HF), and CKD, and previous exposure to insulin. For CKD, in addition to the patient characteristics mentioned above, we further considered histories of retinopathy and neuropathy, and recent use of metformin and acarbose within 90 days before the index date. To ensure the between-group comparability of SGLT2i and DPP4i users in each patient subgroup, the PSM procedure was conducted for each subgroup and the primary analyses were then performed within subgroups.

© 2022 Peng ZY et al. *JAMA Network Open.*

eTable 1. Strengthening the Reporting of Observational Studies in Epidemiology checklist

STROBE Statement—Checklist of items that should be included in reports of ***cohort studies***

	Item No	Recommendation	Page No
**Title and abstract**	1	(*a*) Indicate the study's design with a commonly used term in the title or the abstract	1
		(*b*) Provide in the abstract an informative and balanced summary of what was done and what was found	3-4
**Introduction**			
Background/rationale	2	Explain the scientific background and rationale for the investigation being reported	6-7
Objectives	3	State specific objectives, including any prespecified hypotheses	7
**Methods**			
Study design	4	Present key elements of study design early in the paper	8
Setting	5	Describe the setting, locations, and relevant dates, including periods of recruitment, exposure, follow-up, and data collection	8-9
Participants	6	(*a*) Give the eligibility criteria, and the sources and methods of selection of participants. Describe methods of follow-up	8-9
		(*b*) For matched studies, give matching criteria and number of exposed and unexposed	8
Variables	7	Clearly define all outcomes, exposures, predictors, potential confounders, and effect modifiers. Give diagnostic criteria, if applicable	8-9
Data sources/ measurement	8*	For each variable of interest, give sources of data and details of methods of assessment (measurement). Describe comparability of assessment methods if there is more than one group	8-9
Bias	9	Describe any efforts to address potential sources of bias	10
Study size	10	Explain how the study size was arrived at	N/A
Quantitative variables	11	Explain how quantitative variables were handled in the analyses. If applicable, describe which groupings were chosen and why	9
Statistical methods	12	(*a*) Describe all statistical methods, including those used to control for confounding	9-10

© 2022 Peng ZY et al. *JAMA Network Open.*

		(*b*) Describe any methods used to examine subgroups and interactions	10
		(*c*) Explain how missing data were addressed	N/A
		(*d*) If applicable, explain how loss to follow-up was addressed	N/A
		(*e*) Describe any sensitivity analyses	10
**Results**			
Participants	13*	(a) Report numbers of individuals at each stage of study—eg numbers potentially eligible, examined for eligibility, confirmed eligible, included in the study, completing follow-up, and analysed	11, eFigure1
		(b) Give reasons for non-participation at each stage	N/A
		(c) Consider use of a flow diagram	eFigure1
Descriptive data	14*	(a) Give characteristics of study participants (eg demographic, clinical, social) and information on exposures and potential confounders	11, Table1
		(b) Indicate number of participants with missing data for each variable of interest	N/A
		(c) Summarise follow-up time (eg, average and total amount)	N/A
Outcome data	15*	Report numbers of outcome events or summary measures over time	11, Table2

Main results	16	(*a*) Give unadjusted estimates and, if applicable, confounder-adjusted estimates and their precision (eg, 95% confidence interval). Make clear which confounders were adjusted for and why they were included	11-12, Table2
		(*b*) Report category boundaries when continuous variables were categorized	N/A
		(*c*) If relevant, consider translating estimates of relative risk into absolute risk for a meaningful time period	11
Other analyses	17	Report other analyses done—eg analyses of subgroups and interactions, and sensitivity analyses	12-13
**Discussion**			
Key results	18	Summarise key results with reference to study objectives	14
Limitations	19	Discuss limitations of the study, taking into account sources of potential bias or imprecision. Discuss both direction and magnitude of any potential bias	17
Interpretation	20	Give a cautious overall interpretation of results considering objectives, limitations, multiplicity of analyses, results from similar studies, and	14-16

© 2022 Peng ZY et al. *JAMA Network Open.*

		other relevant evidence	
Generalisability	21	Discuss the generalisability (external validity) of the study results	N/A
**Other information**			
Funding	22	Give the source of funding and the role of the funders for the present study and, if applicable, for the original study on which the present article is based	19

*Give information separately for exposed and unexposed groups.

Note: An Explanation and Elaboration article discusses each checklist item and gives methodological background and published examples of transparent reporting. The STROBE checklist is best used in conjunction with this article (freely available on the Web sites of PLoS Medicine at http://www.plosmedicine.org/, Annals of Internal Medicine at http://www.annals.org/, and Epidemiology at http://www.epidem.com/). Information on the STROBE Initiative is available at http://www.strobe-statement.org.

© 2022 Peng ZY et al. *JAMA Network Open.*

eTable 2. Operational definitions of baseline characteristics defined by ICD-9-CM and ICD-10-CM disease diagnosis codes

**Characteristics**	**ICD-9-CM disease code**	**ICD-10-CM disease codes**
Chronic kidney disease[1]	585	N18
Neuropathy[2,3]	250.6, 354, 355, 356.9, 357.2, 358.1, 713.5, 951.0, 951.1, 951.3	E08.4, E09.4, E10.4, E11.4, E13.4, G60.9, G73.3, H49.0, H49.1, H49.2
Retinopathy[2,3]	250.5, 362.01, 362.02, 361, 362.1, 362.81-362.83, 362.53, 369, 379.23	E08.3, E09.3, E10.3, E11.3, E13.3, H33.0, H54.0, H54.4, H43.1
Peripheral vascular disease[2,3]	250.7, 442.3, 443.81, 443.9, 892.1, 443.9, 444.22, 785.4, 0.4, 707.1	E08.5, E09.5, E10.5, E11.5, E13.5, I72.4, I73.9, I74.3, I70.23-I70.26, A48.0, L97, L98.4, E08.621, E08.622, E09.621, E09.622, E10.621, E10.622, E11.621, E11.622, E13.621, E13.622
Cerebrovascular disease[4,5]	433, 434, 430, 431, 432, 435, V1254	I63, I60, I61, I62, G45
Cardiovascular disease[4-6]	428, 410, 411, 413, 414, V4581, V4582, 4292, 426, 427, 78551, V1253	I50, I21, I25, I2510, I44, I45, I46, I47, I48, I49, R57, Z8674
Heart failure[6]	428	I50
Acute myocardial infarction[5,6]	410	I21
Ischemic heart disease[7,8]	411, 413, 414, V4581, V4582	I25
Diabetic ketoacidosis[2,3]	250.1, 250.2, 250.3	E08.1, E09.1, E10.1, E11.1, E13.1, E08.641, E09.641, E10.641, E11.641, E13.641
Hypoglycemia[9,10]	2508, 2510, 2511, 2512, 9623	E15, E160, E161, E162, E1164, T38.3 (except T38.3X6)

Abbreviations: ICD-9-CM, International Classification of Diseases ninth edition Clinical Modification; ICD-10-CM, International Classification of Diseases tenth edition Clinical Modification.

Note: All variables were measured from outpatient, inpatient, and emergency department files

© 2022 Peng ZY et al. *JAMA Network Open.*

of the National Health Insurance Research Database.

References

1. *Diabetes Obes Metab* 2021;23 Suppl 2:19-27.
2. *Am J Manag Care* 2012;18(11):721-726.
3. *Am J Manag Care* 2019;25(2):e45-e49.
4. *Diabetes Care* 2018;41(5):917-928.
5. *J Epidemiol* 2014;24(6):500-507.
6. *Diabetes Care* 2018;41(5):917-28.
7. *Acta Psychiatr Scand* 2019;140(3):265-274.
8. *J Am Heart Assoc* 2017;6(2).
9. *BMC Endocr Disord* 2008;8:4.
10. *Diabetes Care* 2019;42(4):e58-59.

© 2022 Peng ZY et al. *JAMA Network Open.*

eTable 3. Operational definitions of study outcomes defined by ICD-9-CM and ICD-10-CM disease diagnosis codes and data sources for measurement

Outcomes	ICD-9-CM disease codes	ICD-10-CM disease codes	Data files in NHIRD
Myocardial infarction[1]	410	I21	Inpatient and emergency department
Stroke[2,3]	430, 431-434	I60-I63	Inpatient and emergency department
Heart failure[4]	428	I50	Inpatient and emergency department
Chronic kidney disease[5,6*]	585	N18	Inpatient, emergency, and outpatient department

Abbreviations: ICD-9-CM, International Classification of Diseases ninth edition Clinical Modification; ICD-10-CM, International Classification of Diseases tenth edition Clinical Modification; NHIRD, National Health Insurance Research Database.

[*]Study outcome event of chronic kidney disease (CKD) was confirmed when a patient had at least three consecutive disease diagnoses of CKD within three months, which is supported by the KDIGO guidelines in 2020 and was confirmed by clinical experts.

References

1. *J Epidemiol* 2014;24(6):500-7.
2. *Pharmacoepidemiology and Drug Safety* 2011;20(3):236-42.
3. *J Formos Med Assoc* 2015;114(3):254-9.
4. *Diabetes Care* 2018;41(5):917-28.
5. *Diabetes Obes Metab* 2021;23 Suppl 2:19-27.
6. *Kidney Int* 2020 Oct;98(4S):S1-S115.

© 2022 Peng ZY et al. *JAMA Network Open.*

eTable 4. Baseline characteristics of study cohorts before propensity score matching

Characteristics	Cohort for CVDs in Aim 1			Cohort for CKD in Aim 2		
	DPP4is	SGLT2is	SMD*	DPP4is	SGLT2is	SMD*
Number of subjects	51,808	22,859		45,437	21,658	
Age at index date (years, mean [SD])	64.6 (12.5)	57.2 (11.3)	**-0.62**	63.9 (12.3)	57.1 (11.2)	**-0.62**
Male (%)	53.4	57.2	0.08	52.8	57.0	0.08
Number of GLDs in year before index date (mean [SD])	1.5 (1.0)	1.6 (1.0)	0.09	1.5 (1.0)	1.6 (1.0)	0.09
Duration of diabetes at index date (years, mean [SD])	8.4 (3.2)	8.3 (3.2)	-0.04	8.3 (3.1)	8.3 (3.1)	-0.04
**Surrogate indicators for baseline renal function (%)**						
Participants in pre-ESRD program in year before index date	4.8	4.0	**-0.22**	1.4	0.1	**-0.15**
Metformin prescribed within 90 days before index date	41.7	39.8	-0.04	43.5	39.9	-0.04
Acarbose prescribed within 90 days before index date	9.0	10.9	0.06	8.9	10.9	0.06
**Previous healthcare service utilization in year before index date**						
Number of glycated hemoglobin tests (mean [SD])	5.5 (2.9)	6.1 (2.9)	**0.20**	5.4 (2.8)	6.1 (2.9)	**0.21**
Number of low-density lipoprotein tests (mean [SD])	3.7 (2.5)	4.0 (2.6)	**0.14**	3.6 (2.4)	4.0 (2.6)	**0.14**

© 2022 Peng ZY et al. *JAMA Network Open.*

Subjects who took bone mineral density tests (%)	0.1	1.0	-0.07	1.6	0.9	-0.06
**Diabetes-related complications in year before index date (%)**						
Chronic kidney disease	13.3	5.2	**-0.25**	N/A	N/A	N/A
Neuropathy	9.4	9.1	-0.01	9.1	9.0	-0.01
Retinopathy	8.0	7.7	-0.01	7.5	7.6	-0.01
Peripheral vascular disease	4.2	3.3	-0.05	3.9	3.1	-0.05
Cerebrovascular disease	8.3	4.1	**-0.17**	8.0	4.1	**-0.17**
Cardiovascular disease	19.4	19.3	-0.00	17.8	19.1	-0.00
Heart failure	4.8	3.4	-0.07	3.7	3.2	-0.07
Acute myocardial infarction	1.5	1.8	0.02	1.2	1.8	0.02
Ischemic heart disease	12.0	12.7	0.02	11.2	12.6	0.02
Diabetic ketoacidosis	0.3	0.1	-0.05	0.2	0.1	-0.05
Hypoglycemia	1.6	0.2	**-0.14**	1.2	0.2	**-0.14**
**GLDs prescribed in year before index date (%)**						
Metformin	57.4	53.4	-0.08	59.5	53.5	-0.08
Sulfonylureas	46.3	45.6	-0.02	46.4	45.4	-0.02
Meglitinides	7.8	5.4	**-0.10**	6.8	5.3	**-0.10**
Thiazolidinediones	12.5	17.1	**0.13**	12.6	17.1	**0.13**
Acarbose	14.7	17.6	0.08	14.5	17.6	0.08
GLP1-RAs	0.3	2.1	**0.17**	0.3	2.1	**0.17**
Insulins	13.4	20.8	**0.20**	11.9	20.4	**0.20**
**CVD-related medication history in year before**						

© 2022 Peng ZY et al. *JAMA Network Open.*

**index date (%)**						
Lipid-lowering medications	69.5	79.6	**0.23**	69.2	79.3	**0.23**
Alpha blockers	5.7	4.0	-0.08	4.6	3.8	-0.08
Beta blockers	33.9	33.6	-0.01	32.0	33.3	-0.01
RAAS agents	61.4	61.6	0.01	59.2	61.0	0.01
Diuretics	20.8	13.1	**-0.21**	17.5	12.5	**-0.21**
Calcium channel blockers	37.8	27.5	**-0.22**	35.6	27.1	**-0.22**
Anti-arrhythmics	3.0	2.1	-0.06	2.7	2.0	-0.06
Cardiac glycosides	1.7	1.1	-0.04	1.5	1.1	-0.04
Vasodilators	13.1	12.8	-0.01	11.6	12.6	-0.01
Antiplatelets	38.1	34.8	-0.07	35.9	34.3	-0.07
Anticoagulants	2.7	1.8	-0.06	2.4	1.7	-0.06

Abbreviations: CVD, cardiovascular disease; CKD, chronic kidney disease; DPP4is, dipeptidyl peptidase-4 inhibitors; SGLT2is, sodium-glucose cotransporter-2 inhibitors; SMD, standardized mean difference; SD, standard deviation; GLDs, glucose-lowering drugs; ESRD, end-stage renal disease; GLP1-RAs, glucagon-like peptide 1-receptor agonists; N/A, not applicable; RAAS, renin-angiotensin-aldosterone system.

*An absolute value of SMD greater than 0.1 indicates a statistically significant difference in patient characteristics between drug groups (presented in bold).

© 2022 Peng ZY et al. *JAMA Network Open.*

eTable 5. Results of Cox proportional hazard model analyses on study outcomes among cohorts using either high-dimensional propensity-score or propensity-score weighting procedures (sensitivity analyses)

	Hazard ratio associated with SGLT2i versus DPP4i use (95% CI)				
	Primary analysis	Sensitivity analysis			
	PSM cohort	hdPS-matched cohort	IPTW cohort	Stabilized IPTW cohort	SMRW cohort
**Cardiovascular outcome in Aim 1**					
HHF	0.61 (0.53-0.70)	0.76 (0.60-0.96)	0.60 (0.57-0.64)	0.60 (0.55-0.67)	0.60 (0.53-0.68)
3P-MACE*	0.70 (0.61-0.79)	0.78 (0.68-0.88)	0.71 (0.67-0.76)	0.71 (0.65-0.79)	0.69 (0.61-0.78)
4P-MACE†	0.67 (0.60-0.73)	0.74 (0.67-0.82)	0.67 (0.64-0.71)	0.67 (0.63-0.73)	0.66 (0.60-0.72)
Myocardial infarction	0.71 (0.57-0.90)	0.80 (0.68-0.94)	0.74 (0.66-0.83)	0.74 (0.62-0.89)	0.73 (0.58-0.91)
Stroke	0.69 (0.59-0.80)	0.67 (0.58-0.77)	0.74 (0.68-0.79)	0.74 (0.66-0.83)	0.68 (0.59-0.80)
Cardiovascular death	0.51 (0.35-0.76)	0.59 (0.39-0.88)	0.43 (0.36-0.52)	0.43 (0.32-0.59)	0.48 (0.32-0.70)
All-cause death	0.46 (0.39-0.53)	0.58 (0.50-0.68)	0.43 (0.40-0.45)	0.42 (0.38-0.47)	0.43 (0.37-0.49)
**Kidney outcome in Aim 2**					
CKD	0.38 (0.33-0.43)	0.38 (0.34-0.43)	0.51 (0.49-0.53)	0.51 (0.48-0.55)	0.49 (0.45-0.53)

Abbreviations: SGLT2i, sodium-glucose cotransporter-2 inhibitor; DPP4i, dipeptidyl peptidase-4 inhibitor; CI, confidence interval; PSM, propensity-score matching; hdPS, high-dimensional propensity score; IPTW, inverse probability of treatment weighting; SMRW, Standardized mortality ratio weighting; HHF, hospitalization for heart failure; CKD, chronic kidney disease; 3P-MACE, three-point major adverse cardiovascular event; 4P-MACE, four-point major adverse cardiovascular event.

Notes:

Trimming for propensity scores that were either more than 0.95 or less than 0.05 was done before the estimation of the weights. The weights for generating the IPTW cohort were estimated using the following equations: $Weights_{SGLT2is}$=1/PS and $Weight_{DPP4is}$=1/(1-PS). The weights for generating the stabilized IPTW cohort were estimated using the following equations: $Weights_{SGLT2is}$=Prevalence of SGLT2i users (%)/PS and

© 2022 Peng ZY et al. *JAMA Network Open.*

$Weight_{DPP4is}$= Prevalence of DPP4i users (%)/(1-PS). The weights for generating the SMRW cohort were estimated using the following equations: $Weight_{SGLT2is}$=1 and $Weight_{DPP4is}$=PS/(1-PS). Overall, the hazard ratios of study outcomes associated with SGLT2i versus DPP4i use among pseudo-cohorts from the propensity-score weighting procedures were all statistically significant.

[*]3P-MACE comprised non-fatal myocardial infarction, non-fatal stroke, and cardiovascular death.

[†]4P-MACE comprised hospitalization for heart failure, non-fatal myocardial infarction, non-fatal stroke, and cardiovascular death.

© 2022 Peng ZY et al. *JAMA Network Open.*

eTable 6. Event rate and hazard ratio associated with SGLT2i versus DPP4i use in hospitalization for heart failure outcome (subgroup analyses)

	PS-matched pair	Estimated event rate (events/100 pys)		Estimated HR associated with SGLT2i versus DPP4i use (95% CI)
		SGLT2is	DPP4is	
**Primary analysis**	21,144	1.05	1.72	0.61 (0.53-0.70)
**Subgroup analyses**				
**Age**				
≥ 65 years old	6,000	2.11	2.88	0.73 (0.61-0.88)
< 65 years old	15,046	0.67	1.19	0.56 (0.46-0.68)
**Duration of diabetes**				
≥ 8 years	12,438	1.03	2.16	0.48 (0.40-0.56)
< 8 years	8,734	1.10	1.35	0.81 (0.65-1.01)
**Sex**				
Male	12,007	1.25	1.87	0.66 (0.56-0.78)
Female	9,121	0.82	1.65	0.49 (0.40-0.62)
**History of CVD**				
Yes	4,001	4.23	5.48	0.77 (0.65-0.91)
No	16,992	0.35	0.81	0.43 (0.34-0.55)
**History of HF**				
Yes	690	17.05	22.33	0.77 (0.62-0.95)
No	20,429	0.57	1.15	0.49 (0.41-0.59)
**History of CKD**				
Yes	1,159	2.78	3.83	0.73 (0.50-1.06)
No	19,951	0.95	1.56	0.61 (0.53-0.71)
**Previous exposure to insulin**				
Yes	3,482	1.59	2.86	0.56 (0.43-0.73)
No	17,371	0.97	1.37	0.70 (0.60-0.82)

Abbreviations: PS, propensity score; pys, person-years; HR, hazard ratio; SGLT2is, sodium glucose cotransporter 2 inhibitors; DPP4is, dipeptidyl peptidase 4 inhibitors; CI, confidence interval; CVD, cardiovascular disease; HF, heart failure; CKD, chronic kidney disease.

© 2022 Peng ZY et al. *JAMA Network Open.*

eTable 7. Event rate and hazard ratio associated with SGLT2i versus DPP4i use in chronic kidney disease outcome (subgroup analyses)

	PS-matched pair	Estimated event rate (events/100 pys)		Estimated HR associated with SGLT2i versus DPP4i use (95% CI)
		SGLT2is	DPP4is	
**Primary analysis**	19,951	1.16	3.07	0.38 (0.33-0.43)
**Subgroup analyses**				
**Age**				
≥ 65 years old	5,561	1.74	4.04	0.43 (0.35-0.52)
< 65 years old	14,256	0.90	2.54	0.35 (0.30-0.41)
**Duration of diabetes**				
≥ 8 years	11,644	1.22	3.51	0.35 (0.30-0.40)
< 8 years	8,310	1.02	2.34	0.43 (0.35-0.53)
**Previous exposure to insulin**				
Yes	3180	1.62	4.58	0.35 (0.27-0.46)
No	16,529	1.06	2.70	0.39 (0.34-0.45)
**Sex**				
Male	11,281	1.22	3.36	0.36 (0.31-0.42)
Female	8,659	1.11	2.66	0.42 (0.34-0.51)
**History of CVD**				
Yes	3,741	1.52	3.85	0.39 (0.31-0.51)
No	16,125	1.08	2.88	0.37 (0.32-0.43)
**History of retinopathy**				
Yes	1,517	1.30	6.93	0.19 (0.13-0.28)
No	18,406	1.14	2.67	0.43 (0.38-0.49)
**History of neuropathy**				
Yes	1794	1.52	3.49	0.44 (0.30-0.63)
No	18114	1.11	3.05	0.36 (0.32-0.41)
**Surrogate indicator for renal function**				
Metformin use	7973	1.10	2.26	0.48 (0.39-0.60)
Acarbose use	2153	1.20	4.18	0.29 (0.20-0.41)

Abbreviations: PS, propensity score; pys, person-years; HR, hazard ratio; SGLT2is, sodium glucose cotransporter 2 inhibitors; DPP4is, dipeptidyl peptidase 4 inhibitors; CI, confidence interval; CVD, cardiovascular disease.

Note: Metformin use and acarbose use within 90 days prior to the initiation of SGLT2i or DPP4i therapy were used as proxies for patients with an estimated glomerular filtration rate level of more than 30 and 25 mL/min/1.73 m^2, respectively.

© 2022 Peng ZY et al. *JAMA Network Open.*

eFigure 1. Flowchart of study cohort selection

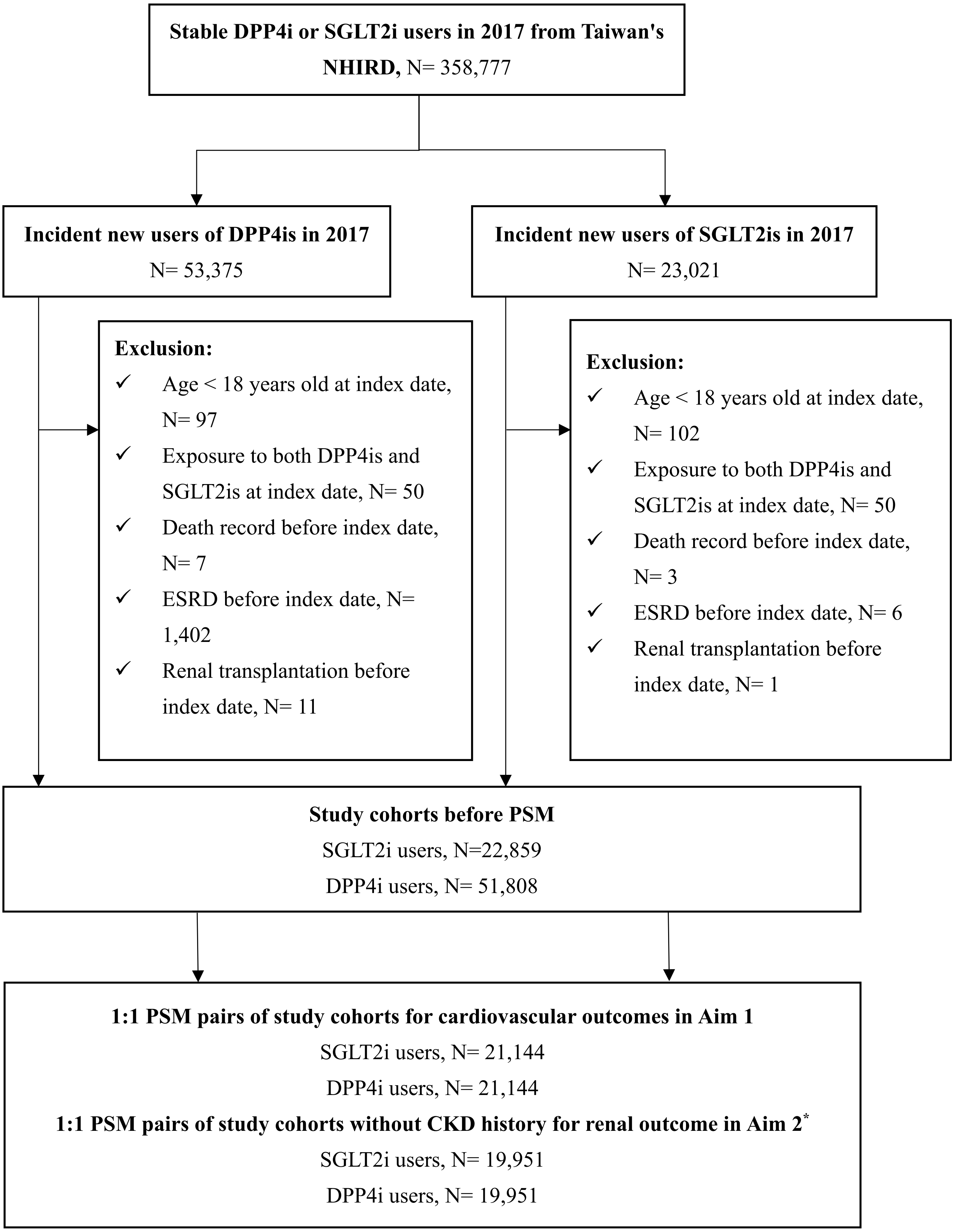

Abbreviations: DPP4i, dipeptidyl peptidase-4 inhibitor; SGLT2i, sodium-glucose cotransporter-2 inhibitor; NHIRD, National Health Insurance Research Database; ESRD,

© 2022 Peng ZY et al. *JAMA Network Open.*

end-stage renal disease; PSM, propensity-score matching; CKD, chronic kidney disease.
*Subjects diagnosed with chronic kidney disease prior to the index date were excluded from the patient population (i.e., 22,859 SGLT2i and 51,808 DPP4i users). Propensity score matching was then performed to generate the study cohort for Aim 2.

© 2022 Peng ZY et al. *JAMA Network Open.*

eFigure 2. Kernel density plots of propensity score for study cohorts (a) before and (b) after propensity-score matching for Aim 1 and those for study cohort (c) before and (d) after propensity-score matching for Aim 2

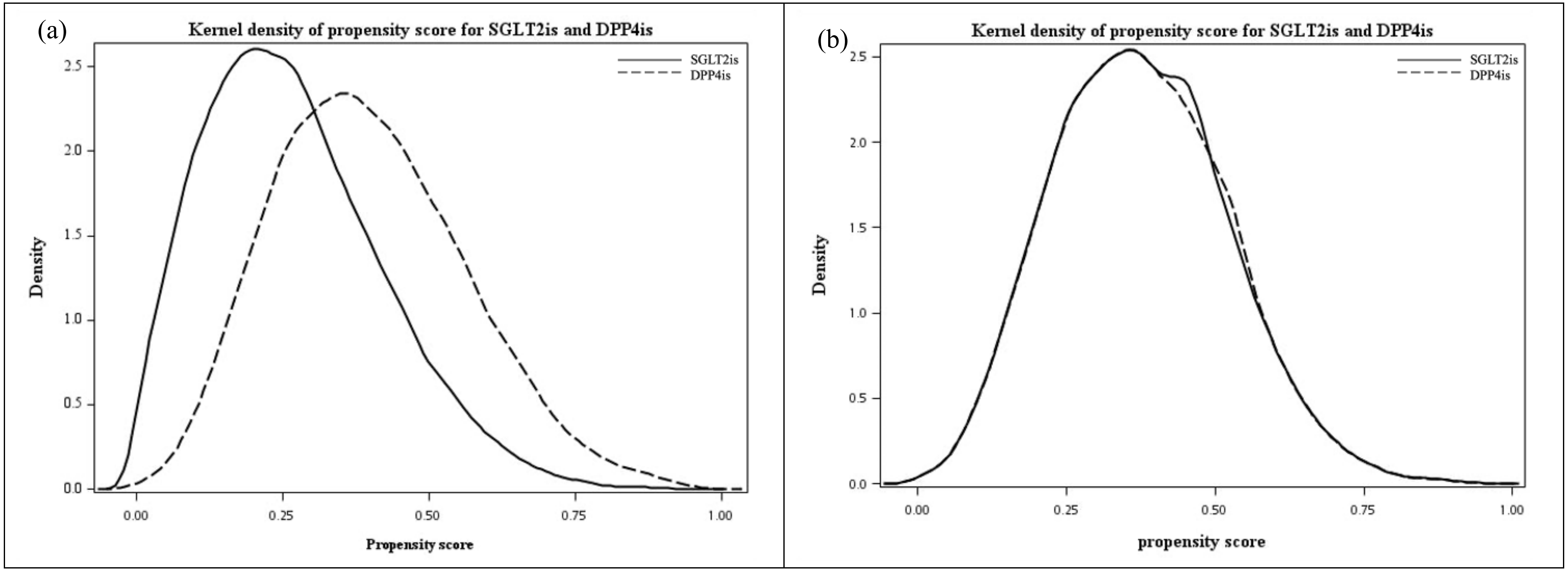

© 2022 Peng ZY et al. *JAMA Network Open.*

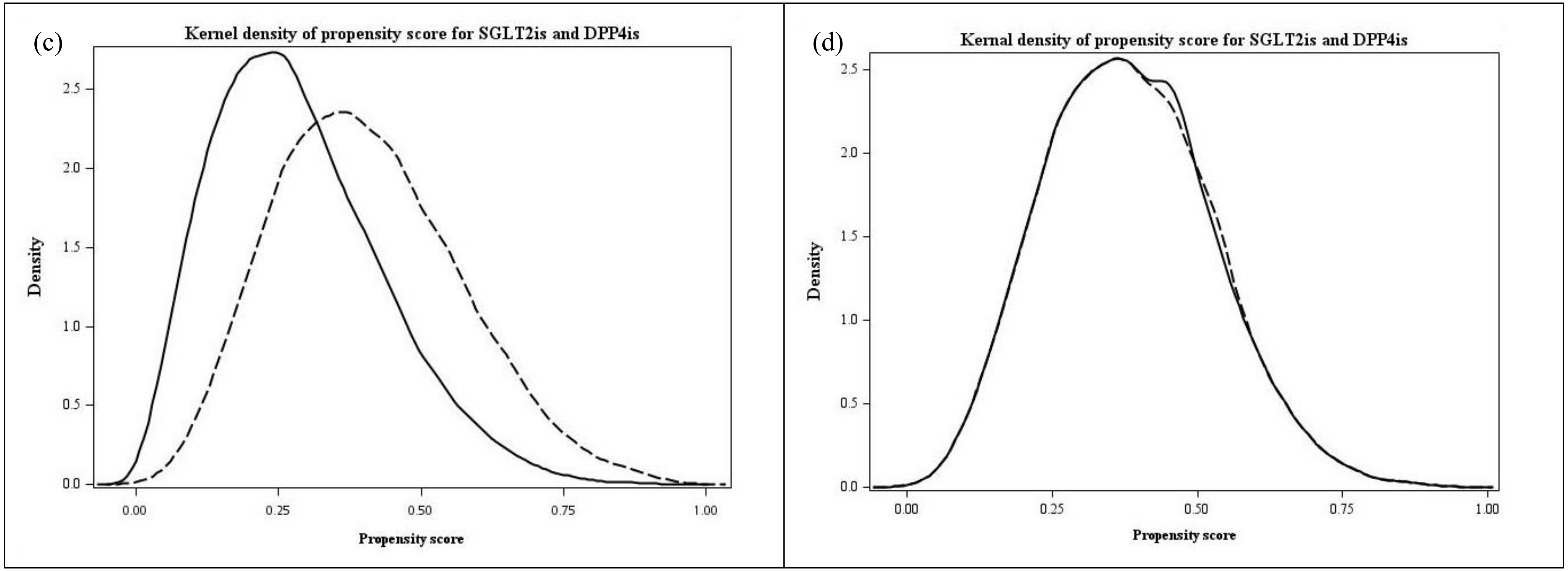

Abbreviations: SGLT2i, sodium-glucose cotransporter-2 inhibitors; DPP4is, dipeptidyl peptidase-4 inhibitors.

Note: (a) propensity score distribution of CVD cohorts in Aim 1 before propensity score matching; (b) propensity score distribution of CVD cohorts in Aim 1 after propensity score matching; (c) propensity score distribution of CKD cohorts in Aim 2 before propensity score matching; (d) propensity score distribution of CKD cohorts in Aim 2 after propensity score matching.

© 2022 Peng ZY et al. *JAMA Network Open.*

eFigure 3. Kaplan-Meier survival curves of SGLT2i and DPP4i users for (a) hospitalization for heart failure, (b) 3P-MACE, (c) 4P-MACE, (d) myocardial infarction, (e) stroke, (f) cardiovascular death, (g) all-cause death, (h) chronic kidney disease, and (i) dental visits for tooth care

(a) Hospitalization for heart failure

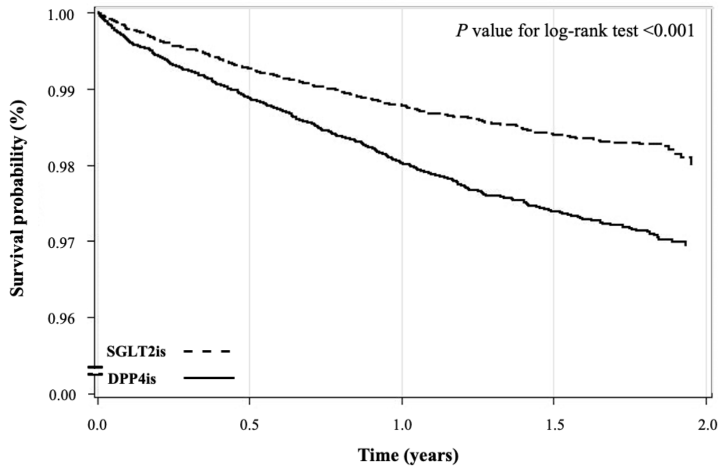

No. at Risk					
SGLT2is	21144	20941	20765	12066	0
DPP4is	21144	20800	20447	12569	0

(b) 3P-MACE*

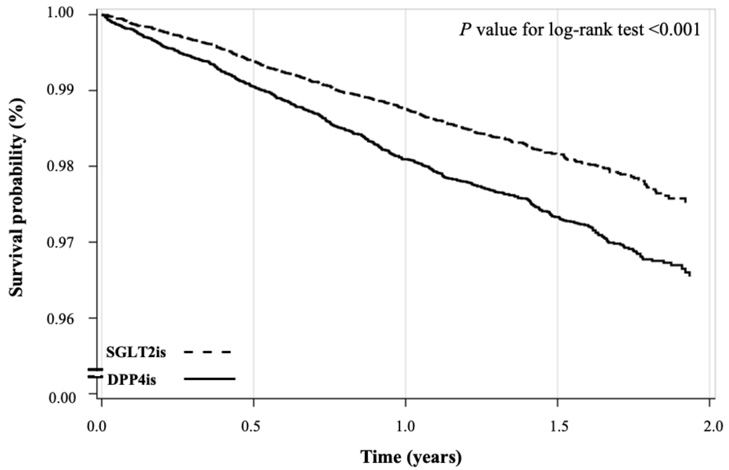

No. at Risk					
SGLT2is	21144	20970	20769	12052	0
DPP4is	21144	20841	20480	12602	0

(c) 4P-MACE†

(d) Myocardial infarction

© 2022 Peng ZY et al. *JAMA Network Open.*

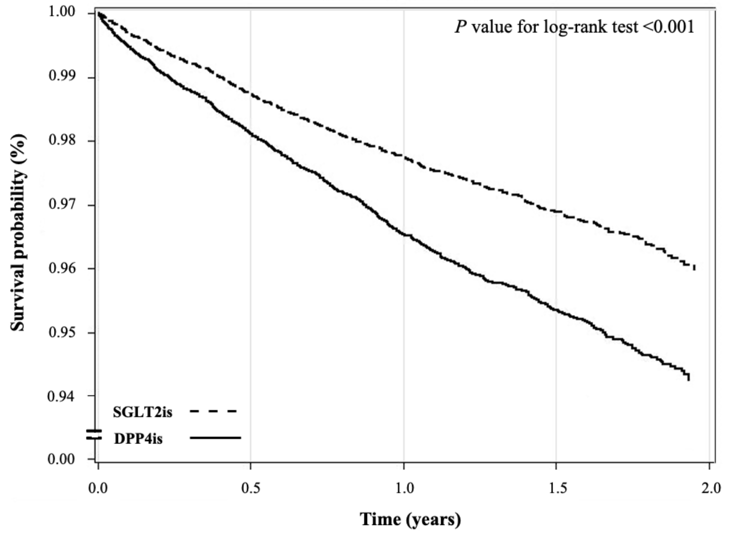

No. at Risk

SGLT2is	21144	20837	20567	11900	0
DPP4is	21144	20654	20180	12366	0

(e) Stroke

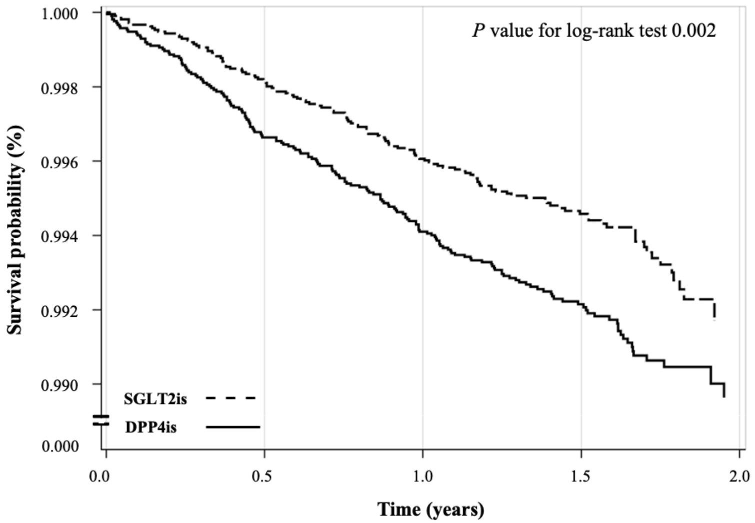

No. at Risk

SGLT2is	21144	21051	20924	12188	0
DPP4is	21144	20956	20701	12776	0

(f) Cardiovascular death

© 2022 Peng ZY et al. *JAMA Network Open.*

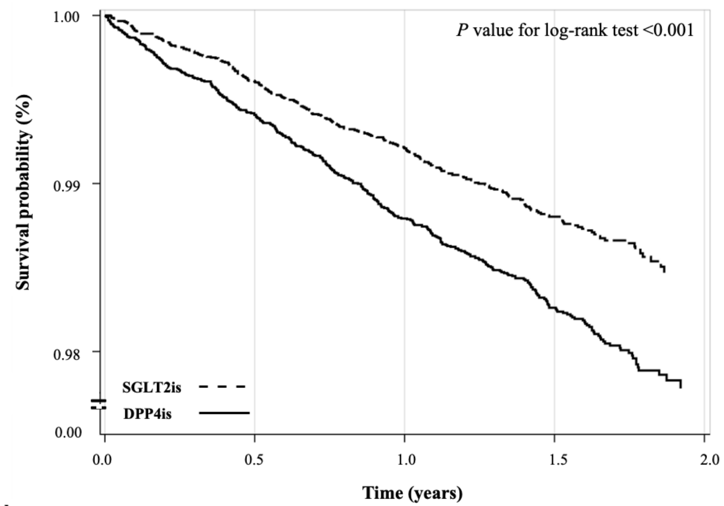

No. at Risk

SGLT2is	21144	21005	20839	12107	0
DPP4is	21144	20905	20580	12676	0

(g) All-cause death

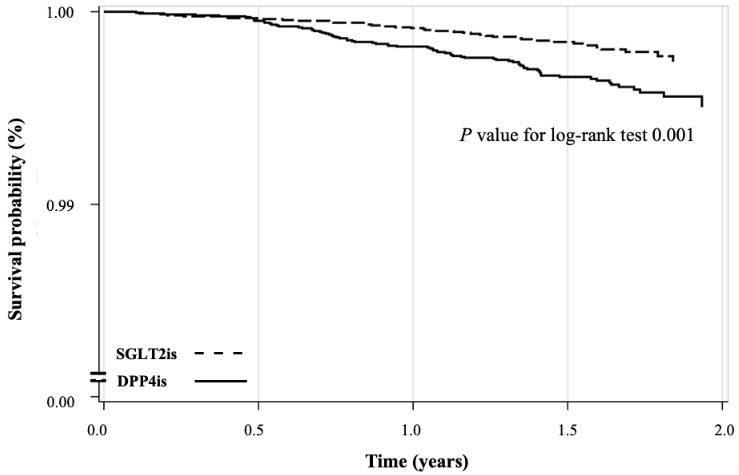

No. at Risk

SGLT2is	21144	21086	20996	12246	0
DPP4is	21144	21024	20806	12858	0

(h) Chronic kidney disease

© 2022 Peng ZY et al. *JAMA Network Open.*

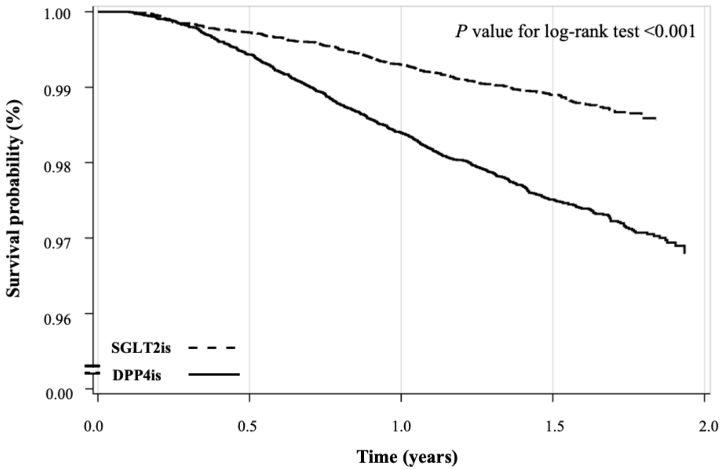

No. at Risk

SGLT2is	21144	21086	20996	12246	0
DPP4is	21144	21024	20806	12858	0

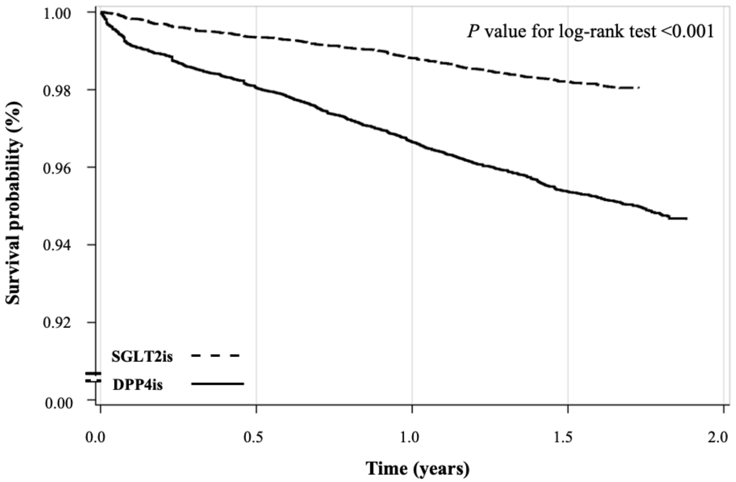

No. at Risk

SGLT2is	19951	19769	19582	11370	0
DPP4is	19951	19451	18994	11589	0

(i) Dental visits for tooth care

© 2022 Peng ZY et al. *JAMA Network Open.*

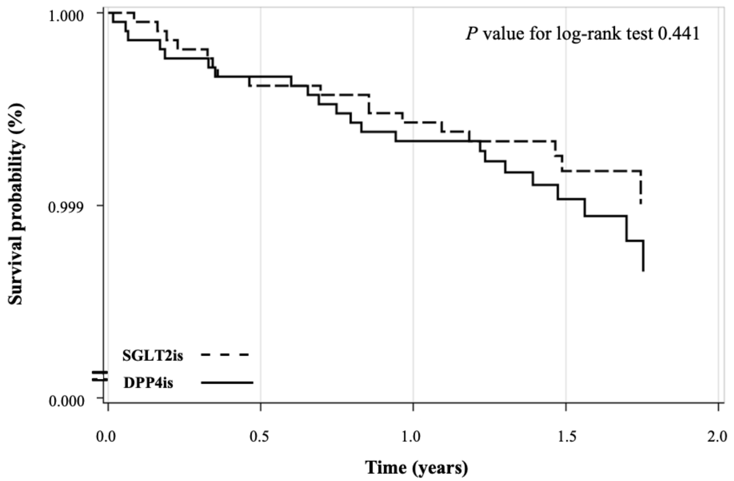

No. at Risk

SGLT2is	21144	21078	20984	12234	0
DPP4is	21144	21017	20792	12844	0

Abbreviations: SGLT2is, sodium-glucose cotransporter-2 inhibitors; DPP4is, dipeptidyl peptidase-4 inhibitors; MACE, major cardiovascular events.

Note:

*3P-MACE comprised non-fatal myocardial infarction, non-fatal stroke, and cardiovascular death.

†4P-MACE comprised hospitalization for heart failure, non-fatal myocardial infarction, non-fatal stroke, and cardiovascular death.

© 2022 Peng ZY et al. *JAMA Network Open.*

eDescription 2. Comparison and interpretations of restricted mean survival time analysis and hazards ratio estimates

Although direct comparison in the magnitude of the treatment effect reported from hazard ratios (HRs) and difference in restricted mean survival time analysis (RMST) may not be feasible under consideration of different calculation scales and time horizons of these measurements (i.e., HR as a relative risk ratio between two treatment groups during a follow-up period since treatment was initiated versus difference in RMST as an absolute time difference between two treatment groups over a prespecified timeframe), difference in RMST may be a relatively conservative estimate for reporting treatment effects, especially in real-world data-based studies, where the HR estimate might be prone to overestimate treatment effects. For example, our HR estimates indicated 22-30% and 42-54% risk reductions in three point-major cardiovascular events (3P-MACE) and all-cause death, respectively, for the use of SGLT2is versus DPP4is in a real-world general T2D population (Tables 2 and 3), whereas 6-18% and 3-33% risk reductions were reported in Kloecker et al.'s study for SGLT2i-associated cardiovascular outcomes trials, where SGLT2is were compared to placebo/standard care and high-cardiovascular-risk patients were targeted. Our difference in RMST results suggest that the postponement for 3P-MACE and all-cause death following the use of SGLT2is versus DPP4is was 2.6-4.1 and 3.6-6.1 days, respectively (Tables 2 and 3), and the postponement was estimated to be 4.1-32.0 and 1.8-23.3 days, respectively, in Kloecker et al.'s study.

© 2022 Peng ZY et al. *JAMA Network Open.*
