# Supplementary material for: Restricted Mean Survival Time Analysis to Estimate SGLT2i–Associated Heterogeneous Treatment Effects on Primary and Secondary Prevention of Cardiorenal Outcomes in Patients With Type 2 Diabetes in Taiwan
Source: JAMA Netw Open. 2022 Dec 15;5(12):e2246928. doi: 10.1001/jamanetworkopen.2022.46928 (PMC9856417; doi:10.1001/jamanetworkopen.2022.46928)
Supplement: Supplement 2. — Data Sharing Statement [file jamanetwopen-e2246928-s002.pdf]

Data Sharing Statement

Peng. Restricted Mean Survival Time Analysis to Estimate SGLT2i-Associated Heterogeneous Treatment Effects on Primary and Secondary Prevention of Cardiorenal Outcomes in Patients With Type 2 Diabetes in Taiwan. *JAMA Netw Open*. Published December 15, 2022. doi:10.1001/jamanetworkopen.2022.46928

Data
Data available: No
